# Plasticity in cell migration modes across development, physiology, and disease

**DOI:** 10.3389/fcell.2024.1363361

**Published:** 2024-04-23

**Authors:** Mona Pourjafar, Vijay K. Tiwari

**Affiliations:** ^1^ Institute of Molecular Medicine, University of Southern Denmark, Odense C, Denmark; ^2^ Wellcome-Wolfson Institute for Experimental Medicine, School of Medicine, Dentistry, and Biomedical Science, Queens University Belfast, Belfast, United Kingdom; ^3^ Danish Institute for Advanced Study (DIAS), Odense M, Denmark; ^4^ Department of Clinical Genetics, Odense University Hospital, Odense C, Denmark

**Keywords:** cell migration, microenvironment, cellular plasticity, cytoskeleton, cell signaling, gene expression

## Abstract

Cell migration is fundamental to both development and adult physiology, including gastrulation, brain development, angiogenesis, wound healing, bone remodeling, tissue homeostasis, and the immune response. Additionally, misguided cellular migration is implicated in disease pathologies such as cancer metastasis and fibrosis. The microenvironment influences cell migration modes such as mesenchymal, amoeboid, lobopodial, and collective, and these are governed through local signaling by affecting the gene expression and epigenetic alteration of migration-related genes. Plasticity in switching between migration modes is essential for key cellular processes across various contexts. Understanding the mechanisms of cell migration modes and its plasticity is essential for unraveling the complexities of this process and revealing its implications in physiological and pathological contexts. This review focuses on different modes of cell migration, including their aberrant migration in disease pathologies and how they can be therapeutically targeted in disease conditions such as cancer.

## Cell migration

**FIGURE 1:**
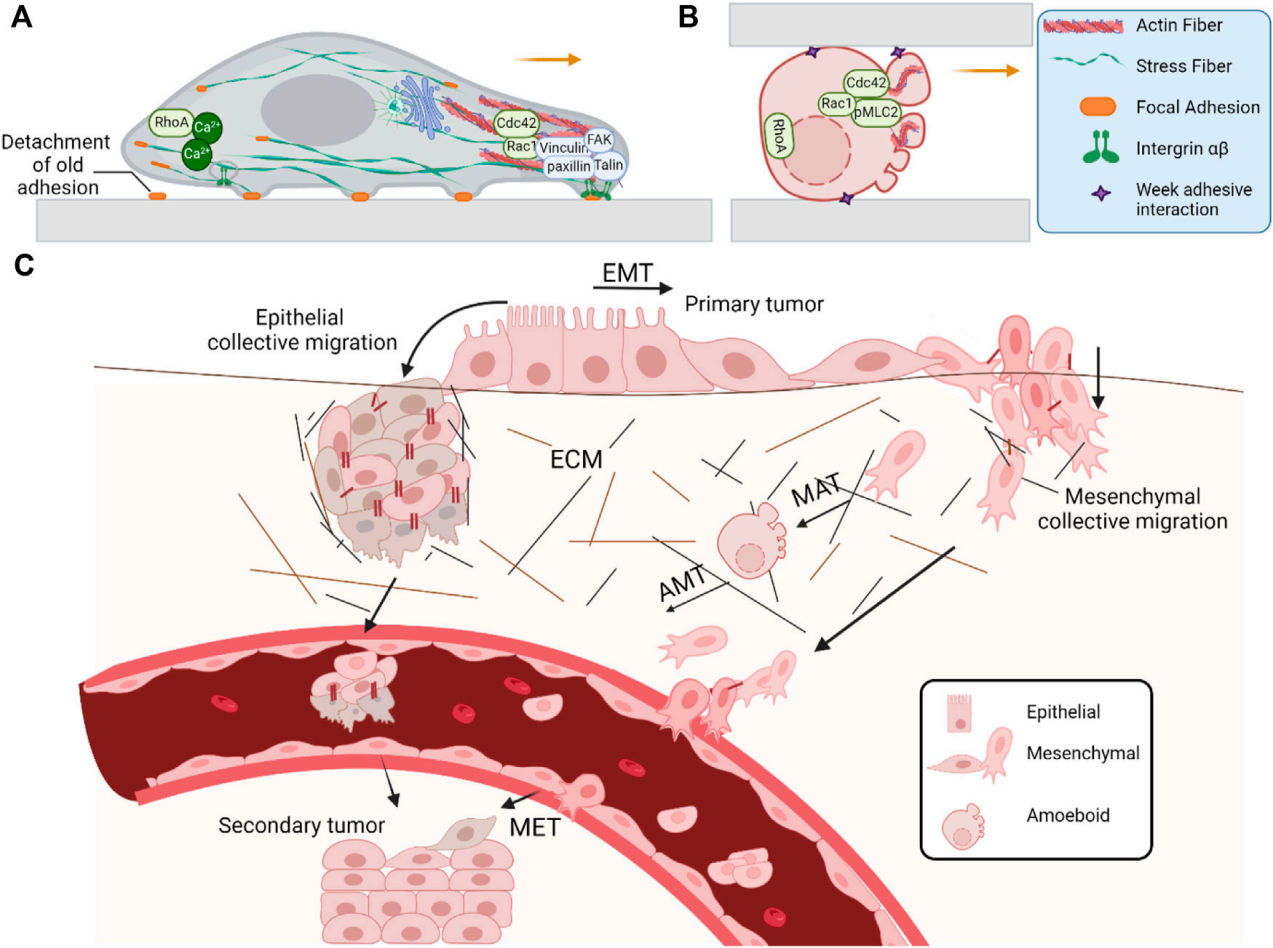
Molecular mechanism of the cell movement during **(A)** mesenchymal migration and **(B)** amoeboid migration. Mesenchymal migration involves the extension of protrusions through actin polymerization at the leading edge, adhesion to the substrate, and forward movement driven by contractile forces mainly at the rear. In amoeboid migration, under confinement**,** cells extend blebs and pseudopods through actin polymerization to adhere to the substrate with a weak adhesive interaction. This mode relies on myosin II sliding along actin filaments at the cell’s rear, generating contraction and squeezing forces that propel the cytoplasm and cell body forward. **(C)** Diversity in cell migration strategies and cellular plasticity; cells can adapt their migration mechanism by reversible transitions such as epithelial–mesenchymal transition (EMT), mesenchymal–epithelial transition (MET), mesenchymal–amoeboid transition (MAT), amoeboid–mesenchymal transition (AMT), epithelial–amoeboid transition (EAT), and single- or collective-cell migration. These adaptive responses are influenced by the environment or cellular properties with alternation of cell–cell adhesions, cell–matrix interactions, cytoskeletal organization, or pericellular proteolysis.

Michael Abercrombie was a pioneer in defining the preliminary concept of cell migration who outlined a comprehensive four-step cycle of cell crawling that requires the coordination of several cellular processes (Trepat et al., 2012). Migration of cells now is more defined and clarified in current research. First, the protrusion of the leading edge occurs, shaping the lamella, lamellipodia, filopodia, and bleb structures through F-actin polarization of cells controlled by the actin-related protein (Arp) 2/3 complex and myosin II. At the leading edge, Rac1/Cdc42 activation promotes actin polymerization, while at the trailing edge, activity of the small GTPase RhoA leads to actomyosin contractions and allows the cell body to move forward (Schaks et al., 2019). Second, the generation of initial adhesions with the substrate occurs through integrins, focal adhesion kinase (FAK), paxillin, talin, and vinculin as the push-off point (Fierro Morales et al., 2022). Third, the contraction of actomyosin stress fibers propels the cell forward through cytoplasm rearrangement and organelle repositioning through RhoA (Kurosaka and Kashina, 2008). Finally, trailing edge retraction enables the cell to advance through detachment of the previous adhesion from the substrate. This process depends on the calcium-dependent protease calpain, which cleaves adhesion proteins including talin, vinculin, and FAK and is involved in the endocytosis of adhesion receptors (Kurosaka and Kashina, 2008; Torres et al., 2018) (Figures 1A, B).

## Cell migration in physiology, development, and disease

Cell migration plays critical roles in a wide array of physiological, developmental, and disease-related processes. In the physiological context, cell migration is involved in embryogenesis, the immune response, and wound healing, while during developmental stages, it is involved in the organization of the nervous system and the generation of specialized organs and tissues. In pathological processes, such as cancer, cell migration leads to acquiring of invasive and migratory capabilities in malignant cells (Kurosaka and Kashina, 2008; SenGupta et al., 2021).

During embryogenesis development, the embryo undergo a highly complex chain of events leading to germ layer formation, positioning, patterning, and organ morphogenesis. Among these events, cell migration plays a crucial role, enabling different lineages of cells to move over short and long distances across the body. The embryonic migratory events occur through the posterior epiblasts, neural crest, and germ cells. During gastrulation a new population of mesenchymal cells is generated from epiblast cells, forming the mesoderm and definitive endoderm layers. Additionally, redistribution of undifferentiated cells and local rearrangements of differentiating cells, neural crest, and germ cell migration assist in heart development, vasculogenesis, and brain development. The majority of these migrations occur after gastrulation (Kurosaka and Kashina, 2008; Saykali et al., 2019). During the embryonic development of the mammalian cortex, neuroepithelial stem cells (also known as apical progenitors) located at the apical boundary of the ventricular zone (VZ) switch to differentiative divisions that result in the generation of either basal progenitors (BPs), which leave the VZ to establish the subventricular zone (SVZ), or neurons. Migrating neurons predominantly appear in the intermediate zone (IZ), while mature neurons organize a six-layered structure in the cortical plate (CP) (Sun and Hevner, 2014). In this regard, NeuroD1, as a bHLH transcription factor, is a highly potent factor that promotes the neuronal fate through binding to regulatory elements of neuronal genes (Pataskar et al., 2016) and, in cooperation with Tcf12, forms a complex to set active chromatin and targets the expression of genes involved in cell migration and cortical development (Singh et al., 2022).

During the development of the immune system, the precursors of immune cells, also known as hematopoietic stem cells, migrate from the bone marrow to the thymus for further differentiation or to specific tissues to settle as tissue sentinel cells. The defense mechanisms of the immune system also rely on the motility capacity of immune cells toward invading pathogens, tissue damage, and primary developing tumor cells under the immune surveillance concept (Vesperini et al., 2021; Delgado et al., 2022). Immune cells mainly use amoeboid movements involving leading edge protrusion, cell body contraction, and weaker adhesive interactions for faster migration (Friedl and BJNi, 2008). They use integrin-mediated adhesion to overcome tissue barriers like the endothelial layer. During three-dimensional migration, immune cells operate independently of integrins, enabling them to navigate swiftly through various organs without needing to adapt to changing extracellular ligands. They use a migration model involving the flow of actin and actomyosin squeezing to facilitate movement (Lammermann et al., 2008). In response to proinflammatory mediators, Th1, Th17, Treg, and possibly Th22 engage in rolling along the endothelial lumen, a process facilitated by selectins and integrins. This is followed by their activation, adhesion to endothelial cells, and extravasation into the inflamed joint. Hence, T-cell migration inhibitors could be a promising approach for anti-inflammatory therapy (Mellado et al., 2015).

Wound healing following an injury necessitates extensive communication between various cellular components and their extracellular matrix occurring in four phases; first, coagulation and hemostasis; second, activation of diverse inflammatory cells; third, proliferation and migration of cells and along with matrix deposition (re-epithelialization); and angiogenesis and eventually remodeling of the extracellular matrix (ECM) and the formation of scar tissue (Baron et al., 2020; Mayya et al., 2021). Wound-associated fibroblasts play a critical role in wound healing by initiating the proliferative phase of repair and replacing the fibrin blood clot with collagen (McDougall et al., 2006). These fibroblasts tend to have a large lamellipodium extending into the wound with few stress fibers in the cell by using growth factors of the wound site including the epidermal growth factor (EGF) and platelet-derived growth factor (PDGF) (Trepat et al., 2012; Addis et al., 2020). During re-epithelialization, cells surrounding the wound move collectively across a temporal matrix abundant in fibrin and fibronectin (Martin, 1997). Keratinocytes become activated by the downregulation of cell adhesion molecules, loss of epithelial cell polarization, and the appearance of an actin-rich leading edge toward the wound area. They migrate by anchorage with new ECM components and releasing metalloproteinase (MMP). During angiogenesis in the proliferative phase, endothelial cells migrate and invade the connective tissue by releasing ECM-tied growth factors, such as fibroblast growth factor (FGF), vascular endothelial growth factor (VEGF), and insulin-like growth factor (IGF) via formation of lamellipodia (Torres et al., 2018).

Bone formation, essential for maintaining bone health throughout life, involves the generation of specialized organs and tissues, as well as bone modeling, remodeling, and repairing fractured bones, which require mature osteoblasts to deposit bone. In bone formation, mesenchymal stem cells (MSCs) differentiate into chondrocytes or osteoblasts but first need to migrate to the bone surface (Su et al., 2018). Therefore, osteoblast precursors derived from circulation or resident stem cell pools must migrate within the three-dimensional bone space to reach the site of bone formation using single- and/or collective-cell movement. During osteogenic differentiation, in the early stages, cell migration speeds up due to activation of Cdc42 and Rac1 Rho-family small GTPases, while in the later stage, migration declines but adhesiveness increases due to the reduction of the phosphorylation level of FAK (Ichida et al., 2011). Additionally, osteoblast migration can be triggered by osteoblast surface receptors mediating cell adhesion and polarization. Furthermore, osteoblast migration can be guided by chemotaxis (the migration of cells toward attractant chemicals) and haptotaxis (directional cell movement in response to adhesive substrates such as the ECM) as landmarks for navigation (Thiel et al., 2018; Thiel et al., 2018).

During the entire cascade of cancer development, especially during the invasion of cancer cells into the surrounding tissue and the vasculature, cell migration occurs, causing severe organ failure. Cancer cell spread can be prompted by tumor microenvironment parameters such as hypoxia, chemoattractants, ECM stiffness, and insufficient nutrient supply. Tumor cells spread in different ways: as individual cells via the mesenchymal mode, which is controlled by cytoskeletal contractility and integrin-mediated ECM adhesion, or through amoeboid modes, where they squeeze through narrow spaces of the ECM in proteolysis-independent ECM remodeling (Figures 1A, B). Additionally, cancer cells can propel as collective groups (Yamaguchi et al., 2005; Novikov et al., 2021; Wu et al., 2021). Furthermore, following the shedding of cancer cells from the primary tumor to the bloodstream, cells can circulate either individually as circulating tumor cells (CTCs) or clusters of CTCs known as circulating tumor microemboli (CTM) that are responsible for tumor metastasis. Although the CTC clusters are less dominant than individual CTCs, they exhibit significantly up to 100-fold higher metastatic potential, which indicates a poor clinical prognosis in specific cancer cases (Aceto et al., 2014; Cheung et al., 2016; Liu et al., 2019) (Figure 1C).

## Single- and collective-cell migration

Cell migrations are classified into single-cell migration and collective-cell migration. Single-cell migration enables cells to move individually toward and between tissue compartments, which are associated with a front–rear polarity and losing some epithelial characteristics, leading to the initiation of an epithelial–mesenchymal transition (EMT) program. Two primary modes of migration are observed during single-cell migration, namely, amoeboid (including blebby and pseudopodal amoeboid) and mesenchymal migration. Amoeboid migration is characterized by the formation of blebs, weak cell–ECM adhesions, and high-velocity movement by passing through the pores of the ECM and fibers in non-proteolytic degradation of the ECM. Mesenchymal migration is marked by robust stress fibers, polarization, and the presence of leading and trailing edges through the expansion of paths by integrin-mediated adhesion to the ECM and proteolytic degradation of the surrounding matrix (Huttenlocher and Horwitz, 2011; Hecht et al., 2015) (Figures 1A, B). Single-cell migration generally occurs in immune cell trafficking, morphogenesis (Aman and Piotrowski, 2010), and wound healing (Sonnemann and Bement, 2011).

Cells may also form cohesive clusters and mobilize collectively. Collective-cell migration refers to the coordinated movement of cell groups, sheets, or chains, in the same direction at a similar speed. This is facilitated by intact cell–cell junctions which enable neighboring cells to adhere and interact with each other that are stabilized by cadherins (e.g., E−, N-, and P-cadherin) while invading the ECM for more persistent migration (Choi et al., 2016). Collective-cell migration plays a crucial role during development and processes such as tissue shaping (Geudens and Gerhardt, 2011) angiogenic tube formation (Andrew and Ewald, 2010), neural crest cell streaming (Shellard and Mayor, 2020), wound healing, and cancer spreading and aggressivity (Cheung and Ewald, 2016). Collective-cell migration can be categorized into epithelial and mesenchymal collective-cell migration (Lintz et al., 2017; Thüroff et al., 2019; Yamada and Sixt, 2019). During epithelial collective-cell migration, stable cell–cell junctions are maintained, whereas during mesenchymal collective-cell migration, transient and dynamic cell adhesion exists between cells, and this migration relies on autocrine/paracrine signaling. These cell–cell interactions are required to mechanically link one cell to another and influence each other’s motility, formation of protrusions, and overall directionality (Theveneau and Mayor, 2013; Scarpa and Mayor, 2016). In collective-cell migration, a group of cells positioned at the front of the supracellular unit generally assumes the role of the leader cells with a contact-free edge and a mesenchymal phenotype (stable lamellipodia or filopodia toward the substrate) in response to external cues to facilitate directed migration. Meanwhile, follower cells located at the rear extend small, transient cryptic lamellipodia (Figure 1C) (De Pascalis and Etienne-Manneville, 2017). Wnt/planar cell polarity (PCP) (VanderVorst et al., 2019), notch-jagged signaling (Cheung et al., 2016), and hypoxia-inducible factor 1 alpha (HIF-1α) signaling (Lehmann et al., 2017) are some defined key signaling pathways that can mediate collective-cell migration during cancer metastasis.

The structural and molecular compositions of the ECM, encompassing dimensions (1, 2, and 3D), density, gap size, stiffness or elasticity, and orientation, influence various cellular behaviors that directly impact cell shape, guidance, and mode of migration. For instance, in the confined ECM, cells tend to adopt a spindle-like shape, whereas larger pore sizes promote cell rounding. Additionally, increased substrate stiffness reinforces cell protrusions at outward edges, while a softer matrix supports cell rounding. Some cell types can individually or collectively tend to migrate toward substrates with greater stiffness, a phenomenon known as durotaxis. Durotaxis can also synergize with chemotaxis (migration of cells toward gradients of chemical factors), effectively aligning the actomyosin machinery of cell groups cooperatively to promote efficient directional collective migration of neural crest cells (Shellard and Mayor, 2021).

Epithelial cell colonies have been observed to respond to substrate stiffness with an increase in several leader cells at the finger-like protrusions with increase in stiffness levels (Balcioglu et al., 2020). Furthermore, mobile cells show a tendency to align structure. External determinants, such as cell–cell interactions (stable or transient junctions, or their absence), cell–matrix adhesions primarily mediated by integrins, and cell protrusion, strongly impact the efficiency and mode of the migration chosen by the cells. Strong cell–substrate adhesions promote cell conversion to an elongated or spread-out morphology in the case of stromal fibroblasts or myoblasts, while moderate- or low-level cell–matrix adhesions encourage the cells to be less elongated and generate smaller lamellipodia and pseudopodia in the case of epithelial cells and leukocytes (Friedl, 2004; Friedl and Wolf, 2010). In addition, the transition between single and collective migration can occur in certain conditions and in response to environmental stimuli. As cell–cell junctions can form *de novo* and resolve again, upregulation of cell–cell adhesion and their aggregation lead to individual-to-collective migration (Wolf et al., 2007). Under hypoxia conditions, collective-cell migration of epithelial cancer can be switched to single-cell amoeboid migration that is driven by HIF-1 and could be mediated by activation of TWIST as an EMT transcription regulator (Lehmann et al., 2017).

## 1-, 2-, and 3-dimensional migration

Micrometer-scale gaps and pores within biological tissues limit cell motion to a single-spatial dimension (1D). In this context, cells migrate through confined environments, including across a line or a narrow linear bundle of collagen fibrillar structures found in the tissue’s interstitial matrix; through pores and channels in the ECM; and in blood/lymph capillaries (Vesperini et al., 2021). The 1D ECM constrains cell shape by preventing lateral cell spreading and promoting a unidirectional phenotype. Cells display more rapid migration in the 1D conditions due to more efficient protrusion of the leading edge relying on myosin IIA (MIIA) actomyosin contractility (Doyle et al., 2009; Doyle et al., 2012). Cell migration along a 1D substrate can closely mimic the biological characteristics of cells such as uniaxial individual cell morphology, posterior orientation of the centrosome, reliance on non‐muscle myosin II, and non-dependency of the migration rate to matrix ligand density in the 3D matrix (Doyle et al., 2009). On the other hand, 2D migration is an extensively studied cell migration, which involves cells adhering to a substrate on one side of flat surfaces such as glass or plastic *in vitro* (Nourshargh and Alon, 2014). However, the anti-migratory effects of pharmacological agents in unconfined 2D substrates to confined environments (3D) are often ineffective. *In vivo*, 2D cell surfaces include the peritoneum covering all internal organs, the pleura covering the lungs and thorax wall, ventricles of the brain, and inner surfaces of larger blood and lymph vessels that form unobstructed channels containing interstitial fluid and glycosaminoglycans (Friedl and Alexander, 2011). In the process of immune cell extravasation, the rolling, firm attachment, and intravascular crawling of these cells along the walls of blood vessels resemble a 2D migration pattern (Nourshargh and Alon, 2014; Vesperini et al., 2021). On 2D surfaces, cells display multi-axial phenotypes, with a centrosome located in front of the nucleus, and myosin II restrains cell migration speed (Yamada et al., 2019). In 2D migration, cells necessitate unilateral adhesion to the substrate, which provides stable enough but transient attachment. In 3D migration, the most common migration mode through tissues, cells squeeze through complex or dense extracellular structures in a confinement environment that occurs during the early stages of development, immune cell surveillance and defense, wound healing, and cancer invasion. In 3D migration, the interwoven network of collagen fibers imposes space limitations against the moving cell body, leading to characteristic changes in their morphology. Mesenchymal, amoeboid, and lobopodial are the major categories of 3D cell migration, which use different types of cell protrusions. In mesenchymal migration, cells establish robust attachments to the ECM through focal adhesions linked to mature stress fibers. These cells exhibit polarized organization of the cytoskeleton in the direction of migration, and the centrosome is in front of the nucleus with a high capacity for degrading the matrix. In contrast, cells with amoeboid migration lose the adhesions and protease activity and form contraction-based blebs or actin-driven protrusions to move smoothly on the substrate (Figure 1B). The organelle relocation positions the centrosome behind the nucleus during amoeboid migration. Based on the fluid flow velocity inside the cytoplasm and the pore pressure, tightly adherent cells also can gain intermediary lobopodial type of migration with exhibition of low protease activity and high RhoA-ROCK-MyoII contractility to form bleb-like blunt protrusions called lobopodia (Seetharaman and Etienne-Manneville, 2020). Hence, the matrix composition, expression, and localization of integrins in cell–matrix adhesions, pore size to rigidity, and elastic behavior can switch cell migration modes through the RhoA–ROCK–myosin-II signaling axis within the 3D ECM. The cell with more diffuse localization of integrins and increased actomyosin contractility undergoes pseudopodia retraction and cell rounding, while the normal integrin expression and function lead to an elongated lobopodia shape (Petrie and Yamada, 2012; Petrie and Yamada, 2012).

## Cell plasticity and migration

Cell plasticity as a fundamental feature of human biology is the ability of cells to reprogram and change their phenotypes and adopt different identities in response to external stimuli, particularly when tissue homeostasis is perturbed as a mechanism of tissue adaptation or regeneration (Mills et al., 2019; Yuan et al., 2019). This phenomenon is relevant to developmental and tissue-specific stem cells, regenerative medicine, and some pathological conditions, particularly neoplasms which are associated with increased plasticity. In this regard, cell plasticity can be displayed by stem cell differentiation (generation of progenitors by stem cell division), dedifferentiation (reversion of mature cells into stem cell state), transdifferentiation (conversion of mature cells into another mature cell lineage), transdetermination (transformation of a progenitor cell into another type of progenitor cell), and palingenesis (re-entry of mature cells to the cell cycle) (Tata et al., 2021; Pérez-González et al., 2023). In the process of migration, cells can switch from different motility modes depending on their context and environmental conditions, which is referred to as migratory plasticity. During the EMT, epithelial cells lose the epithelial characteristics including cell–cell and cell–matrix adhesion and polarity, while acquiring a mesenchymal phenotype (with a cadherin switch), which allows them to switch from a non-motile epithelial to a motile mesenchymal state and initiate migration to a destined location. At the destined location, these mesenchymal-like cells can regain their epithelial phenotype by undergoing a mesenchymal–epithelial transition (MET) in a binary process. Meanwhile, the existence of cells with a series of intermediate states and hybrid epithelial/mesenchymal phenotypes can be demonstrated (Orgaz et al., 2014; Haerinck et al., 2023). Increasing evidences suggest that EMT can contribute to cellular diversity during development and adulthood. Examples of cells using EMT include the neural crest cells in embryos, fibroblasts/mesenchymal cells in injured tissues, and metastatic epithelial cancer cells (Kalluri, 2009; Sahu et al., 2022). Plasticity of cancer cell migration also can be observed as a mesenchymal–amoeboid and amoeboid–mesenchymal through a balance between Rac and Rho signaling and surface protease activity (Wolf et al., 2007; Friedl and Wolf, 2010; Orgaz et al., 2014; Tognoli et al., 2021) and epithelial–amoeboid transition (Choi et al., 2016) targeting RHO/ROCK signaling pathways (Figure 1C). Nevertheless, the evidence shows that both naturally and experimentally induced modifications can cause significant adaptive responses that alter the migration mode instead of complete elimination of migration (Friedl and Wolf, 2010). Cell plasticity is promoted by cell-intrinsic factors, such as DNA damage, mutations in oncogenic and/or tumor-suppressor genes (likely derived from elevated lineage plasticity), deregulation, or aberrant expression of specific genes like homeobox genes, reversible epigenetic alterations, and senescence. Additionally, cell-extrinsic factors and environmental cues including the tumor microenvironment, extracellular matrix, collagen deposition, and matrix stiffness contribute to this dynamic process and induce adaptations by cancer cells for invasion, spread, and resistance to cytotoxic drugs that govern cancer cell plasticity (Poli et al., 2018; Zhang and Goodrich, 2022; Saha et al., 2023). The core EMT-related transcription factors such as Snail (also known as SNAI1), Slug (also known as SNAI2), twist-related protein 1 (TWIST1), zinc-finger E-box-binding homeobox 1 (ZEB1), and ZEB2 proteins (Stemmler et al., 2019) cooperate to downregulate the expression of epithelial markers such as E-cadherin, claudins, and occludin and upregulation of mesenchymal markers such as N-cadherin, vimentin, and fibronectin (Ye and Weinberg, 2015). Apart from the core transcriptional factors, newly identified reprogramming transcription factors such as PRKRIR, ETV6, SRF, HOXB9, TSC22D1, TCF12, NR1H4, PCBP4, NFIL3, MEOX2, HNF4A, SFPI1, LMO7, and CDX2 have been discovered to explain the complexity of changes during cancerous and neuronal EMT (Sahu et al., 2015). Epigenetic plasticity, such as dysregulation of DNA methylation patterns on both tumor suppressors and oncogenes and histone posttranslational modifications (e.g., methylation, acetylation, ubiquitination, phosphorylation, and SUMOylation), enables cancer cells to adapt to exogenous stresses, including conventional and targeted therapies during their progression toward malignancy. Instances of epigenetic modification contributing to permissive chromatin, in association with factors from the tumor microenvironment and the EMT factor, include inactivation of CDH1 by hypermethylation (Graff et al., 1995); maintenance of the CDH1 promoter in the methylated status in assistance with ZEB1 by recruiting DNMT1 (Fukagawa et al., 2015); hyperactivation of TWIST due to binding of MMSET to TWIST promoter and increased methylation at H3K36m2 (Poli et al., 2018); recruitment of histone deacetylases (HDAC1 and HDAC2) and the co-repressor Sin3A to the CDH1 promoter by SNAIL, resulting in the deacetylation of histones H3 and H4 (Serrano-Gomez et al., 2016); trimethylation of histone H3 at lysine 27 for several EMT-associated genes by Ezh2 in a Sox4-dependent manner (Tiwari et al., 2013); and ubiquitination of C-terminal-binding protein1 (CtBP1) through FBXO32 to promote its nuclear retention, which is essential for induction of CtBP1 target genes to create a proper microenvironment for EMT progression (Sahu et al., 2017). As previously mentioned, the phenotypic plasticity of the cell, especially in cancer, can be strongly influenced by cues from the tumor microenvironment. This environment comprises transformed cells and the surrounding normal tissue, tumor-associated macrophages, tumor-associated neutrophils, cancer-associated fibroblasts, and tumor-associated endothelial cells. A variety of tumor cell types release osteopenia and CXCL12/SDF-1 which mobilize stem cells from the bone marrow and facilitate their contribution to the formation of blood vessels within the tumor and promoting tumor growth (Orimo et al., 2005; McAllister et al., 2008). Tumor-associated macrophages (TANs) also known as the M2 phenotype contribute to this regulation by secreting granulocyte–macrophage colony-stimulating factor (GM-CSF) (Su et al., 2014), IL-6, along with tumor necrosis factor-alpha (TNF-α), IL-1β (Guo et al., 2019), and matrix metalloproteases (MMPs) by activation of PI3Kγ, STAT, KLF6, ZEB1, and NFAT1 transcription factors which support tumorigenesis and metastasis, while macrophage plasticity is beneficial during the wound healing process (Ricketts et al., 2021). Neutrophil diversity and plasticity are the dual functions of TANs in the tumor microenvironment and homeostatic conditions. Interferon-β (IFNβ) or a combination of IFNγ and GM-CSF directs neutrophils toward an antitumor state, whereas TGFβ polarizes neutrophil function in a pro-tumor direction (Jaillon et al., 2020). Tumor-associated neutrophils by secreting the cytokine tissue inhibitor matrix metalloproteinase 1 (TIMP-1) induce breast cancer EMT and can also remodel the extracellular matrix via neutrophil collagenase (MMP8) and gelatinase B (MMP9) (Giese et al., 2019; Wang et al., 2019). Cancer-associated fibroblasts (CAFs) originate from tissue-resident fibroblasts and mesenchymal stromal cells. They become activated by factors such as TGFβ, PDGF, and FGF2. These cells are involved in the regulation of the TME through the secretion of activin A (Valenti et al., 2017), FGF5, and the production of fibrillar collagen (Cazet et al., 2018), which supports and promotes the expansion of cancer stem cells and self-renewal of cancer stem cells. CAFs additionally enhance the deposition and remodeling of the ECM, cellular and tissue tension and stiffness, and adjust the tissue structure (Zhang et al., 2023). The tumor-associated endothelial cells also play a part in TME regulation by secreting TGF-β1, periostin, and epoxy eicosanoids to help cancer cells escape from dormancy (Panigrahy et al., 2012; Ghajar et al., 2013) along with vascular endothelial growth factor (VEGF) and CXCR7 to confer tumors with aggressive and lethal properties (Maishi and Hida, 2017).

## Targeting cell migration for the treatment of diseases

Previous successes in targeting cell migration for the treatment of diseases, particularly in cancer therapeutics, are notable. Researchers have developed drugs that specifically inhibit the migration of cancer cells, impeding their ability to spread and metastasize. Key molecules involved in cell migration, such as G-protein-coupled receptors (GPCR), integrins, adhesion proteins (e.g., selectin and cadherin), enzymes (e.g., MMPs), the cytoskeleton, and chemokine/cytokine receptors, serve as targets for drugs that have shown promise in limiting disease progression (Gattringer et al., 2023). Among these, notable examples are the FDA-approved drugs such as the taxane family of drugs (e.g., paclitaxel and cabazitaxel) by targeting the tubulin in cancer cells (Sousa-Pimenta et al., 2023), natalizumab by targeting α4b1 and α4b7 integrins in T cells of the inflamed tissue (Mackay, 2008), plerixafor as a small-molecule CXCR4 antagonist to mobilize hematopoietic stem cell migration to the bloodstream (DiPersio et al., 2009), vedolizumab as an anti-α4β7 antibody for the treatment of inflammatory bowel disease by selective inhibition of lymphocyte trafficking in the intestine (Jovani and Danese, 2013), and lifitegrast, a small molecule targeting αLβ2, used for the treatment of dry eye disease by preventing T-cell activation and release of inflammatory mediators, thus inhibiting the inflammatory pathways (Abidi et al., 2016). Although efforts to develop MMP inhibitors have not shown clinical efficacy, one possible reason for this could be the independence of cell migration modes from structural matrix remodeling, similar to what is observed during amoeboid migration (Raudenská et al., 2023). Similar observations have been made regarding Rho/ROCK inhibitors such as Y27632, which impairs amoeboid-like invasion by restoring cell-surface expression of α2β1-integrin, enhancing FAK autophosphorylation, and potentially promoting mesenchymal invasion (Chang et al., 2018). Antagonists targeting CXCR4, CCR2, CCR5, and CXCR1/2 were also initially developed to disrupt tumor invasiveness. Yet they have also been found to impact immune cell migration within the TME. Despite this, the efforts to develop small molecules and antibodies for targeting these chemokines and their receptor persist (Kohli et al., 2022). For instance, the small-molecule inhibitors of CCR4, including FLX475 and RPT193, have been developed, and FLX475 monotherapy results in beneficial changes in the TME toward a phenotype associated with the response to anti-PD-1/anti-PD-L1 (Brockstedt et al., 2023).

However, the future potential for treating diseases by targeting cell migration is underlined by various strategies. These include using genomics to recognize distinct genetic or molecular signatures linked to abnormal cell migration, exploring molecular mechanisms to reveal new essential migration targets, and increasing therapeutic outcomes through the combination of drugs targeting diverse aspects of cell migration mechanisms or by combining anti-migration strategies with other conventional treatments.

In summary, cell motility is essential for development and life, yet it is also intricately linked to various pathological processes, including cancer metastasis. During cell migration, cells depend on their phenotype and the properties of the surrounding microenvironment (such as the type of protrusion, contractility, internal (mainly nuclear) reorientation, proteolytic capacity, cell–cell, and cell–extracellular matrix adhesions). The modulation of these factors influences the ability of cells to transition between different modes of migration, highlighting the importance of plasticity in the intricate process of cell migration and emphasizing its potential for future treatments of human diseases, including cancer and inflammatory disorder.

## References

[B1] Abidi A. Shukla P. Ahmad A. (2016). Lifitegrast: a novel drug for treatment of dry eye disease. J. Pharmacol. Pharmacother. 7 (4), 194–198. 10.4103/0976-500X.195920 28163544 PMC5242036

[B2] Aceto N. Bardia A. Miyamoto D. T. Donaldson M. C. Wittner B. S. Spencer J. A. (2014). Circulating tumor cell clusters are oligoclonal precursors of breast cancer metastasis. Cell 158 (5), 1110–1122. 10.1016/j.cell.2014.07.013 25171411 PMC4149753

[B3] Addis R. Cruciani S. Santaniello S. Bellu E. Sarais G. Ventura C. (2020). Fibroblast proliferation and migration in wound healing by phytochemicals: evidence for a novel synergic outcome. Int. J. Med. Sci. 17 (8), 1030–1042. 10.7150/ijms.43986 32410832 PMC7211158

[B4] Aman A. Piotrowski T. (2010). Cell migration during morphogenesis. Dev. Biol. 341 (1), 20–33. 10.1016/j.ydbio.2009.11.014 19914236

[B5] Andrew D. J. Ewald A. J. (2010). Morphogenesis of epithelial tubes: insights into tube formation, elongation, and elaboration. Dev. Biol. 341 (1), 34–55. 10.1016/j.ydbio.2009.09.024 19778532 PMC2854166

[B6] Balcioglu H. E. Balasubramaniam L. Stirbat T. V. Doss B. L. Fardin M.-A. Mège R.-M. (2020). A subtle relationship between substrate stiffness and collective migration of cell clusters. Soft Matter 16 (7), 1825–1839. 10.1039/c9sm01893j 31970382

[B7] Baron J. M. Glatz M. Proksch E. (2020). Optimal support of wound healing: new Insights. Dermatology 236 (6), 593–600. 10.1159/000505291 31955162

[B8] Brockstedt D. G. Grant A. Adamik J. Trujillo D. Goyal R. K. Ho W. (2023). “Clinical and biological activity of FLX475, an oral CCR4 antagonist,” in Advanced cancer (American Society of Clinical Oncology).

[B9] Cazet A. S. Hui M. N. Elsworth B. L. Wu S. Z. Roden D. Chan C.-L. (2018). Targeting stromal remodeling and cancer stem cell plasticity overcomes chemoresistance in triple negative breast cancer. Nat. Commun. 9 (1), 2897. 10.1038/s41467-018-05220-6 30042390 PMC6057940

[B10] Chang F. Zhang Y. Mi J. Zhou Q. Bai F. Xu X. (2018). ROCK inhibitor enhances the growth and migration of BRAF‐mutant skin melanoma cells. Cancer Sci. 109 (11), 3428–3437. 10.1111/cas.13786 30168234 PMC6215891

[B11] Cheung K. J. Ewald A. J. (2016). A collective route to metastasis: seeding by tumor cell clusters. Science 352 (6282), 167–169. 10.1126/science.aaf6546 27124449 PMC8183671

[B12] Cheung K. J. Padmanaban V. Silvestri V. Schipper K. Cohen J. D. Fairchild A. N. (2016). Polyclonal breast cancer metastases arise from collective dissemination of keratin 14-expressing tumor cell clusters. Proc. Natl. Acad. Sci. 113 (7), E854–E863. 10.1073/pnas.1508541113 26831077 PMC4763783

[B13] Choi P.-W. Yang J. Ng S.-K. Feltmate C. Muto M. G. Hasselblatt K. (2016). Loss of E-cadherin disrupts ovarian epithelial inclusion cyst formation and collective cell movement in ovarian cancer cells. Oncotarget 7 (4), 4110–4121. 10.18632/oncotarget.6588 26684027 PMC4826193

[B14] Delgado M. G. Lennon-Duménil A.-M. J. F. C. Biology D. (2022). How cell migration helps immune sentinels. Front. Cell Dev. Biol. 10, 1994. 10.3389/fcell.2022.932472 PMC9577558 36268510

[B15] De Pascalis C. Etienne-Manneville S. (2017). Single and collective cell migration: the mechanics of adhesions. Mol. Biol. cell 28 (14), 1833–1846. 10.1091/mbc.E17-03-0134 28684609 PMC5541834

[B16] DiPersio J. F. Stadtmauer E. A. Nademanee A. Micallef I. N. Stiff P. J. Kaufman J. L. (2009). Plerixafor and G-CSF versus placebo and G-CSF to mobilize hematopoietic stem cells for autologous stem cell transplantation in patients with multiple myeloma. Blood, J. Am. Soc. Hematol. 113 (23), 5720–5726. 10.1182/blood-2008-08-174946 19363221

[B17] Doyle A. D. Kutys M. L. Conti M. A. Matsumoto K. Adelstein R. S. Yamada K. M. (2012). Micro-environmental control of cell migration–myosin IIA is required for efficient migration in fibrillar environments through control of cell adhesion dynamics. J. cell Sci. 125 (9), 2244–2256. 10.1242/jcs.098806 22328520 PMC3367941

[B18] Doyle A. D. Wang F. W. Matsumoto K. Yamada K. M. (2009). One-dimensional topography underlies three-dimensional fibrillar cell migration. J. cell Biol. 184 (4), 481–490. 10.1083/jcb.200810041 19221195 PMC2654121

[B19] Fierro Morales J. C. Xue Q. Roh-Johnson M. (2022). An evolutionary and physiological perspective on cell-substrate adhesion machinery for cell migration. Front. Cell Dev. Biol. 10, 943606. 10.3389/fcell.2022.943606 36092727 PMC9453864

[B20] Friedl P. (2004). Prespecification and plasticity: shifting mechanisms of cell migration. Curr. Opin. cell Biol. 16 (1), 14–23. 10.1016/j.ceb.2003.11.001 15037300

[B21] Friedl P. Alexander S. (2011). Cancer invasion and the microenvironment: plasticity and reciprocity. Cell 147 (5), 992–1009. 10.1016/j.cell.2011.11.016 22118458

[B22] Friedl P. Bjni W. (2008). Interstitial leukocyte migration and immune function. Nat. Immunol. 9 (9), 960–969. 10.1038/ni.f.212 18711433

[B23] Friedl P. Wolf K. (2010). Plasticity of cell migration: a multiscale tuning model. J. Cell Biol. 188 (1), 11–19. 10.1083/jcb.200909003 19951899 PMC2812848

[B24] Fukagawa A. Ishii H. Miyazawa K. Saitoh M. (2015). δEF1 associates with DNMT1 and maintains DNA methylation of the E‐cadherin promoter in breast cancer cells. Cancer Med. 4 (1), 125–135. 10.1002/cam4.347 25315069 PMC4312126

[B25] Gattringer J. Gruber C. W. Hellinger R. (2023). Peptide modulators of cell migration: overview, applications and future development. Drug Discov. Today 28, 103554. 10.1016/j.drudis.2023.103554 36921670 PMC7615922

[B26] Geudens I. Gerhardt H. (2011). Coordinating cell behaviour during blood vessel formation. Development 138 (21), 4569–4583. 10.1242/dev.062323 21965610

[B27] Ghajar C. M. Peinado H. Mori H. Matei I. R. Evason K. J. Brazier H. (2013). The perivascular niche regulates breast tumour dormancy. Nat. cell Biol. 15 (7), 807–817. 10.1038/ncb2767 23728425 PMC3826912

[B28] Giese M. A. Hind L. E. Huttenlocher A. (2019). Neutrophil plasticity in the tumor microenvironment. Blood, J. Am. Soc. Hematol. 133 (20), 2159–2167. 10.1182/blood-2018-11-844548 PMC6524564 30898857

[B29] Graff J. R. Herman J. G. Lapidus R. G. Chopra H. Xu R. Jarrard D. F. (1995). E-cadherin expression is silenced by DNA hypermethylation in human breast and prostate carcinomas. Cancer Res. 55 (22), 5195–5199. 7585573

[B30] Guo L. Cheng X. Chen H. Chen C. Xie S. Zhao M. (2019). Induction of breast cancer stem cells by M1 macrophages through Lin-28B-let-7-HMGA2 axis. Cancer Lett. 452, 213–225. 10.1016/j.canlet.2019.03.032 30917918

[B31] Haerinck J. Goossens S. Berx G. (2023). The epithelial–mesenchymal plasticity landscape: principles of design and mechanisms of regulation. Nat. Rev. Genet. 24, 590–609. 10.1038/s41576-023-00601-0 37169858

[B32] Hecht I. Bar-El Y. Balmer F. Natan S. Tsarfaty I. Schweitzer F. (2015). Tumor invasion optimization by mesenchymal-amoeboid heterogeneity. Sci. Rep. 5 (1), 10622. 10.1038/srep10622 26013062 PMC4650638

[B33] Huttenlocher A. Horwitz A. R. (2011). Integrins in cell migration. Cold Spring Harb. Perspect. Biol. 3 (9), a005074. 10.1101/cshperspect.a005074 21885598 PMC3181029

[B34] Ichida M. Yui Y. Yoshioka K. Tanaka T. Wakamatsu T. Yoshikawa H. (2011). Changes in cell migration of mesenchymal cells during osteogenic differentiation. FEBS Lett. 585 (24), 4018–4024. 10.1016/j.febslet.2011.11.014 22100295

[B35] Jaillon S. Ponzetta A. Di Mitri D. Santoni A. Bonecchi R. Mantovani A. (2020). Neutrophil diversity and plasticity in tumour progression and therapy. Nat. Rev. Cancer 20 (9), 485–503. 10.1038/s41568-020-0281-y 32694624

[B36] Jovani M. Danese S. (2013). Vedolizumab for the treatment of IBD: a selective therapeutic approach targeting pathogenic a4b7 cells. Curr. drug targets 14 (12), 1433–1443. 10.2174/13894501113146660206 23980911

[B37] Kalluri R. (2009). EMT: when epithelial cells decide to become mesenchymal-like cells. J. Clin. Invest 119 (6), 1417–1419. 10.1172/JCI39675 19487817 PMC2689122

[B38] Kohli K. Pillarisetty V. G. Kim T. S. (2022). Key chemokines direct migration of immune cells in solid tumors. Cancer gene Ther. 29 (1), 10–21. 10.1038/s41417-021-00303-x 33603130 PMC8761573

[B39] Kurosaka S. Kashina A. (2008). Cell biology of embryonic migration. Birth Defects Res. Part C Embryo Today Rev. 84 (2), 102–122. 10.1002/bdrc.20125 PMC2542983 18546335

[B40] Lammermann T. Bader B. L. Monkley S. J. Worbs T. Wedlich-Soldner R. Hirsch K. (2008). Rapid leukocyte migration by integrin-independent flowing and squeezing. Nature 453 (7191), 51–55. 10.1038/nature06887 18451854

[B41] Lehmann S. Te Boekhorst V. Odenthal J. Bianchi R. van Helvert S. Ikenberg K. (2017). Hypoxia induces a HIF-1-dependent transition from collective-to-amoeboid dissemination in epithelial cancer cells. Curr. Biol. 27 (3), 392–400. 10.1016/j.cub.2016.11.057 28089517

[B42] Lintz M. Muñoz A. Reinhart-King C. A. (2017). The mechanics of single cell and collective migration of tumor cells. J. Biomech. Eng. 139 (2), 0210051–0210059. 10.1115/1.4035121 27814431 PMC5395914

[B43] Liu X. Taftaf R. Kawaguchi M. Chang Y.-F. Chen W. Entenberg D. (2019). Homophilic CD44 interactions mediate tumor cell aggregation and polyclonal metastasis in patient-derived breast cancer models. Cancer Discov. 9 (1), 96–113. 10.1158/2159-8290.CD-18-0065 30361447 PMC6328322

[B44] Mackay C. R. (2008). Moving targets: cell migration inhibitors as new anti-inflammatory therapies. Nat. Immunol. 9 (9), 988–998. 10.1038/ni.f.210 18711436

[B45] Maishi N. Hida K. (2017). Tumor endothelial cells accelerate tumor metastasis. Cancer Sci. 108 (10), 1921–1926. 10.1111/cas.13336 28763139 PMC5623747

[B46] Martin P. J. S. (1997). Wound healing--aiming for perfect skin regeneration. Science 276 (5309), 75–81. 10.1126/science.276.5309.75 9082989

[B47] Mayya C. Kharbhanda S. Haque A. Bhatia DJWHRCT Directions F. Mechanisms of collective cell migration in wound healing: physiology and disease. 2021:55–74.

[B48] McAllister S. S. Gifford A. M. Greiner A. L. Kelleher S. P. Saelzler M. P. Ince T. A. (2008). Systemic endocrine instigation of indolent tumor growth requires osteopontin. Cell 133 (6), 994–1005. 10.1016/j.cell.2008.04.045 18555776 PMC4121664

[B49] McDougall S. Dallon J. Sherratt J. PjptotRSAM M. Physical S. E. (2006). Fibroblast migration and collagen deposition during dermal wound healing: mathematical modelling and clinical implications. Math. Phys. Eng. Sci. 364 (1843), 1385–1405. 10.1098/rsta.2006.1773 16766351

[B50] Mellado M. Martinez-Munoz L. Cascio G. Lucas P. Pablos J. L. Rodríguez-Frade J. M. (2015). T cell migration in rheumatoid arthritis. Front. Immunol. 6, 384. 10.3389/fimmu.2015.00384 26284069 PMC4515597

[B51] Mills J. C. Stanger B. Z. Sander M. (2019). Nomenclature for cellular plasticity: are the terms as plastic as the cells themselves? EMBO J. 38 (19), e103148. 10.15252/embj.2019103148 31475380 PMC6769377

[B52] Nourshargh S. Alon R. (2014). Leukocyte migration into inflamed tissues. Immunity 41 (5), 694–707. 10.1016/j.immuni.2014.10.008 25517612

[B53] Novikov N. M. Zolotaryova S. Y. Gautreau A. M. Denisov E. V. (2021). Mutational drivers of cancer cell migration and invasion. Br. J. cancer 124 (1), 102–114. 10.1038/s41416-020-01149-0 33204027 PMC7784720

[B54] Orgaz J. L. Pandya P. Dalmeida R. Karagiannis P. Sanchez-Laorden B. Viros A. (2014). Diverse matrix metalloproteinase functions regulate cancer amoeboid migration. Nat. Commun. 5, 4255. 10.1038/ncomms5255 24963846 PMC4118761

[B55] Orimo A. Gupta P. B. Sgroi D. C. Arenzana-Seisdedos F. Delaunay T. Naeem R. (2005). Stromal fibroblasts present in invasive human breast carcinomas promote tumor growth and angiogenesis through elevated SDF-1/CXCL12 secretion. Cell 121 (3), 335–348. 10.1016/j.cell.2005.02.034 15882617

[B56] Panigrahy D. Edin M. L. Lee C. R. Huang S. Bielenberg D. R. Butterfield C. E. (2012). Epoxyeicosanoids stimulate multiorgan metastasis and tumor dormancy escape in mice. J. Clin. investigation 122 (1), 178–191. 10.1172/JCI58128 PMC3248288 22182838

[B57] Pataskar A. Jung J. Smialowski P. Noack F. Calegari F. Straub T. (2016). NeuroD1 reprograms chromatin and transcription factor landscapes to induce the neuronal program. EMBO J. 35 (1), 24–45. 10.15252/embj.201591206 26516211 PMC4718003

[B58] Pérez-González A. Bévant K. Blanpain C. (2023). Cancer cell plasticity during tumor progression, metastasis and response to therapy. Nat. Cancer 4 (8), 1063–1082. 10.1038/s43018-023-00595-y 37537300 PMC7615147

[B59] Petrie R. J. Yamada K. M. (2012). At the leading edge of three-dimensional cell migration. J. Cell Sci. 125 (24), 5917–5926. 10.1242/jcs.093732 23378019 PMC4067260

[B60] Poli V. Fagnocchi L. Zippo A. (2018). Tumorigenic cell reprogramming and cancer plasticity: interplay between signaling, microenvironment, and epigenetics. Stem cells Int. 2018, 1–16. 10.1155/2018/4598195 PMC5954911 29853913

[B61] Raudenská M. Petrláková K. Juriňáková T. Fialová J. L. Fojtů M. Jakubek M. (2023). Engine shutdown: migrastatic strategies and prevention of metastases. Trends Cancer 9, 293–308. 10.1016/j.trecan.2023.01.001 36804341

[B62] Ricketts T. D. Prieto-Dominguez N. Gowda P. S. Ubil E. (2021). Mechanisms of macrophage plasticity in the tumor environment: manipulating activation state to improve outcomes. Front. Immunol. 12, 642285. 10.3389/fimmu.2021.642285 34025653 PMC8139576

[B63] Saha S. Pradhan N. Neha B. Mahadevappa R. Minocha S. Kumar S. (2023). “Cancer plasticity: investigating the causes for this agility,” Seminars in cancer biology (Elsevier). 10.1016/j.semcancer.2022.12.005 36584960

[B64] Sahu S. K. Agirre E. Inayatullah M. Mahesh A. Tiwari N. Lavin D. P. (2022). A complex epigenome-splicing crosstalk governs epithelial-to-mesenchymal transition in metastasis and brain development. Nat. cell Biol. 24 (8), 1265–1277. 10.1038/s41556-022-00971-3 35941369

[B65] Sahu S. K. Garding A. Tiwari N. Thakurela S. Toedling J. Gebhard S. (2015). JNK‐dependent gene regulatory circuitry governs mesenchymal fate. EMBO J. 34 (16), 2162–2181. 10.15252/embj.201490693 26157010 PMC4557668

[B66] Sahu S. K. Tiwari N. Pataskar A. Zhuang Y. Borisova M. Diken M. (2017). FBXO32 promotes microenvironment underlying epithelial-mesenchymal transition via CtBP1 during tumour metastasis and brain development. Nat. Commun. 8 (1), 1523. 10.1038/s41467-017-01366-x 29142217 PMC5688138

[B67] Saykali B. Mathiah N. Nahaboo W. Racu M.-L. Hammou L. Defrance M. (2019). Distinct mesoderm migration phenotypes in extra-embryonic and embryonic regions of the early mouse embryo. eLife 8, e42434. 10.7554/eLife.42434 30950395 PMC6450669

[B68] Scarpa E. Mayor R. (2016). Collective cell migration in development. J. Cell Biol. 212 (2), 143–155. 10.1083/jcb.201508047 26783298 PMC4738384

[B69] Schaks M. Giannone G. Rottner K. (2019). Actin dynamics in cell migration. Essays Biochem. 63 (5), 483–495. 10.1042/EBC20190015 31551324 PMC6823167

[B70] Seetharaman S. Etienne-Manneville S. (2020). Cytoskeletal crosstalk in cell migration. Trends cell Biol. 30 (9), 720–735. 10.1016/j.tcb.2020.06.004 32674938

[B71] SenGupta S. Parent C. A. Bear J. E. (2021). The principles of directed cell migration. Nat. Rev. Mol. Cell Biol. 22 (8), 529–547. 10.1038/s41580-021-00366-6 33990789 PMC8663916

[B72] Serrano-Gomez S. J. Maziveyi M. Alahari S. K. (2016). Regulation of epithelial-mesenchymal transition through epigenetic and post-translational modifications. Mol. cancer 15 (1), 18–14. 10.1186/s12943-016-0502-x 26905733 PMC4765192

[B73] Shellard A. Mayor R. (2020). Rules of collective migration: from the wildebeest to the neural crest. Philos. Trans. R. Soc. Lond B Biol. Sci. 375 (1807), 20190387. 10.1098/rstb.2019.0387 32713298 PMC7423382

[B74] Shellard A. Mayor R. (2021). Collective durotaxis along a self-generated stiffness gradient *in vivo* . Nature 600 (7890), 690–694. 10.1038/s41586-021-04210-x 34880503

[B75] Singh A. Mahesh A. Noack F. Cardoso de Toledo B. Calegari F. Tiwari V. K. (2022). Tcf12 and NeuroD1 cooperatively drive neuronal migration during cortical development. Development 149 (3), dev200250. 10.1242/dev.200250 35147187 PMC8918803

[B76] Sonnemann K. J. Bement W. M. (2011). Wound repair: toward understanding and integration of single-cell and multicellular wound responses. Annu. Rev. cell Dev. Biol. 27, 237–263. 10.1146/annurev-cellbio-092910-154251 21721944 PMC4878020

[B77] Sousa-Pimenta M. Estevinho L. M. Szopa A. Basit M. Khan K. Armaghan M. (2023). Chemotherapeutic properties and side-effects associated with the clinical practice of terpene alkaloids: paclitaxel, docetaxel, and cabazitaxel. Front. Pharmacol. 14, 1157306. 10.3389/fphar.2023.1157306 37229270 PMC10203197

[B78] Stemmler M. P. Eccles R. L. Brabletz S. Brabletz T. (2019). Non-redundant functions of EMT transcription factors. Nat. cell Biol. 21 (1), 102–112. 10.1038/s41556-018-0196-y 30602760

[B79] Su P. Tian Y. Yang C. Ma X. Wang X. Pei J. (2018). Mesenchymal stem cell migration during bone formation and bone diseases therapy. Int. J. Mol. Sci. 19 (8), 2343. 10.3390/ijms19082343 30096908 PMC6121650

[B80] Su S. Liu Q. Chen J. Chen J. Chen F. He C. (2014). A positive feedback loop between mesenchymal-like cancer cells and macrophages is essential to breast cancer metastasis. Cancer cell 25 (5), 605–620. 10.1016/j.ccr.2014.03.021 24823638

[B81] Sun T. Hevner R. F. (2014). Growth and folding of the mammalian cerebral cortex: from molecules to malformations. Nat. Rev. Neurosci. 15 (4), 217–232. 10.1038/nrn3707 24646670 PMC4107216

[B82] Tata A. Chow R. D. Tata P. R. (2021). Epithelial cell plasticity: breaking boundaries and changing landscapes. EMBO Rep. 22 (7), e51921. 10.15252/embr.202051921 34096150 PMC8256290

[B83] Theveneau E. Mayor R. (2013). Collective cell migration of epithelial and mesenchymal cells. Cell. Mol. Life Sci. 70, 3481–3492. 10.1007/s00018-012-1251-7 23314710 PMC11113167

[B84] Thiel A. Reumann M. K. Boskey A. Wischmann J. von Eisenhart-Rothe R. Mayer-Kuckuk P. (2018). Osteoblast migration in vertebrate bone. Biol. Rev. Camb. Philosophical Soc. 93 (1), 350–363. 10.1111/brv.12345 PMC6218945 28631442

[B85] Thüroff F. Goychuk A. Reiter M. Frey E. (2019). Bridging the gap between single-cell migration and collective dynamics. Elife 8, e46842. 10.7554/eLife.46842 31808744 PMC6992385

[B86] Tiwari N. Tiwari V. K. Waldmeier L. Balwierz P. J. Arnold P. Pachkov M. (2013). Sox4 is a master regulator of epithelial-mesenchymal transition by controlling Ezh2 expression and epigenetic reprogramming. Cancer cell 23 (6), 768–783. 10.1016/j.ccr.2013.04.020 23764001

[B87] Tognoli M. L. Vlahov N. Steenbeek S. Grawenda A. M. Eyres M. Cano-Rodriguez D. (2021). RASSF1C oncogene elicits amoeboid invasion, cancer stemness, and extracellular vesicle release via a SRC/Rho axis. Embo J. 40 (20), e107680. 10.15252/embj.2021107680 34532864 PMC8521318

[B88] Torres P. Castro M. Reyes M. Torres V. J. (2018). Histatins, wound healing, and cell migration. Oral Dis. 24 (7), 1150–1160. 10.1111/odi.12816 29230909

[B89] Trepat X. Chen Z. Jacobson K. (2012). Cell migration. Compr. Physiol. 2 (4), 2369–2392. 10.1002/cphy.c110012 23720251 PMC4457291

[B90] Valenti G. Quinn H. M. Heynen G. J. Lan L. Holland J. D. Vogel R. (2017). Cancer stem cells regulate cancer-associated fibroblasts via activation of hedgehog signaling in mammary gland tumors. Cancer Res. 77 (8), 2134–2147. 10.1158/0008-5472.CAN-15-3490 28202523

[B91] VanderVorst K. Dreyer C. A. Konopelski S. E. Lee H. Ho H.-Y. H. Carraway I. I. I. K. L. (2019). Wnt/PCP signaling contribution to carcinoma collective cell migration and metastasis. Cancer Res. 79 (8), 1719–1729. 10.1158/0008-5472.CAN-18-2757 30952630 PMC6467734

[B92] Vesperini D. Montalvo G. Qu B. Lautenschläger F. (2021). Characterization of immune cell migration using microfabrication. Biophys. Rev. 13 (2), 185–202. 10.1007/s12551-021-00787-9 34290841 PMC8285443

[B93] Wang Y. Chen J. Yang L. Li J. Wu W. Huang M. (2019). Tumor-contacted neutrophils promote metastasis by a CD90-TIMP-1 juxtacrine–paracrine loop. Clin. Cancer Res. 25 (6), 1957–1969. 10.1158/1078-0432.CCR-18-2544 30482778

[B94] Wolf K. Wu Y. I. Liu Y. Geiger J. Tam E. Overall C. (2007). Multi-step pericellular proteolysis controls the transition from individual to collective cancer cell invasion. Nat. cell Biol. 9 (8), 893–904. 10.1038/ncb1616 17618273

[B95] Wu J.-s. Jiang J. Chen B.-j. Wang K. Tang Y.-l. Liang X.-J. (2021). Plasticity of cancer cell invasion: patterns and mechanisms. Transl. Oncol. 14 (1), 100899. 10.1016/j.tranon.2020.100899 33080522 PMC7573380

[B96] Yamada K. M. Collins J. W. Cruz Walma D. A. Doyle A. D. Morales S. G. Lu J. (2019). Extracellular matrix dynamics in cell migration, invasion and tissue morphogenesis. Int. J. Exp. Pathol. 100 (3), 144–152. 10.1111/iep.12329 31179622 PMC6658910

[B97] Yamada K. M. Sixt M. (2019). Mechanisms of 3D cell migration. Nat. Rev. Mol. cell Biol. 20 (12), 738–752. 10.1038/s41580-019-0172-9 31582855

[B98] Yamaguchi H. Wyckoff J. Jjcoicb C. (2005). Cell migration in tumors. Curr. Opin. cell Biol. 17 (5), 559–564. 10.1016/j.ceb.2005.08.002 16098726

[B99] Ye X. Weinberg R. A. (2015). Epithelial–mesenchymal plasticity: a central regulator of cancer progression. Trends cell Biol. 25 (11), 675–686. 10.1016/j.tcb.2015.07.012 26437589 PMC4628843

[B100] Yuan S. Norgard R. J. Stanger B. Z. (2019). Cellular plasticity in cancer. Cancer Discov. 9 (7), 837–851. 10.1158/2159-8290.CD-19-0015 30992279 PMC6606363

[B101] Zhang L. Goodrich D. W. (2022). RB1, cancer lineage plasticity, and therapeutic resistance. Annu. Rev. Cancer Biol. 6, 201–221. 10.1146/annurev-cancerbio-070120-092840

[B102] Zhang W. Wang J. Liu C. Li Y. Sun C. Wu J. (2023). Crosstalk and plasticity driving between cancer-associated fibroblasts and tumor microenvironment: significance of breast cancer metastasis. J. Transl. Med. 21 (1), 827. 10.1186/s12967-023-04714-2 37978384 PMC10657029

